# Acute Suppurative Thyroiditis in an Intravenous Drug User with a Preexisting Goiter

**DOI:** 10.1155/2018/5098712

**Published:** 2018-03-26

**Authors:** Nikhil Yegya-Raman, Tabitha Copeland, Payal Parikh

**Affiliations:** Department of Medicine, Rutgers Robert Wood Johnson Medical School, New Brunswick, NJ 08901, USA

## Abstract

Acute suppurative thyroiditis (AST) is an uncommon, potentially life-threatening cause of a rapidly enlarging neck mass. It may present similarly to subacute thyroiditis, a relatively benign and self-limiting condition. We report a case of AST in an adult intravenous (IV) drug user with a preexisting goiter who presented with a left forearm abscess that grew methicillin-sensitive *Staphylococcus aureus*. In this particular case, clinical suspicion for AST was high. As a result, early IV antibiotic therapy was initiated, and this led to rapid clinical improvement furthermore preventing airway compromise. To our knowledge, this is the first case of AST in the literature resulting from likely hematogenous spread in the setting of IV drug use and a preexisting goiter. Overall, this case highlights the importance of assessing risk factors for AST in patients whose presentations may seem more typical of subacute thyroiditis. Such an approach will lead to timely diagnosis and treatment to avoid potentially devastating consequences.

## 1. Introduction

Acute suppurative thyroiditis (AST) is a rare but potentially lethal endocrine condition, representing just 0.1–0.7% of all thyroid disease [1]. The rarity of AST is thought to be influenced by several protective trophic factors and anatomic barriers specific to the thyroid gland, including the presence of iodine, hydrogen peroxide, lymphatic drainage, and a thyroid capsule. These mechanisms have been suggested to provide a shield against the development of thyroid suppuration [2–4].

AST is most commonly encountered in the pediatric and adolescent populations, usually in those with congenital anomalies such as a pyriform sinus fistula [5, 6]. Only 8% of cases occur in adulthood; in many cases, the source of infection is not obvious [2, 7]. It may present similarly to subacute thyroiditis, with painful thyroid swelling, fever, and leukocytosis following an upper respiratory tract infection (URTI). AST exhibits a wide range of illness severity, from complete resolution back to a euthyroid state to a greater than 12% mortality rate if no interventions are initiated [2, 3].

Given the high mortality rate without intervention, early diagnosis and treatment of AST remain paramount in order to prevent poor outcomes. There are limited recommendations in the current published literature for a streamlined diagnostic approach and management strategy to provide guidance to clinicians [2, 8, 9]. In this case report, we highlight a diagnostic approach and management of AST by use of targeted intravenous (IV) antibiotics followed by transition to oral antibiotics in an adult patient with a history of IV drug use and a preexisting goiter.

## 2. Case

The patient is a 26-year-old female with a past medical history significant for IV drug use and a right-sided multinodular goiter who presented with five days of anterior neck swelling, predominantly on the right side, and associated tenderness to palpation. In the week prior to presentation, the patient had developed a sore throat, fatigue, and rhinorrhea, along with an abscess on the posterior aspect of her left forearm where she injects heroin. She denied ever injecting drugs into her neck or shoulders. Her review of systems was negative for fevers, chills, rigors, shortness of breath, wheezing, difficulty breathing, dysphagia, and chest pain. She further denied weakness, muscle twitching, diarrhea, constipation, weight loss, weight gain, and palpitations. Her multinodular goiter had been discovered about one year prior. At the time, she was asymptomatic and thyroid function tests were within normal limits, but no fine-needle aspiration (FNA) biopsy was performed.

**Figure 1:**
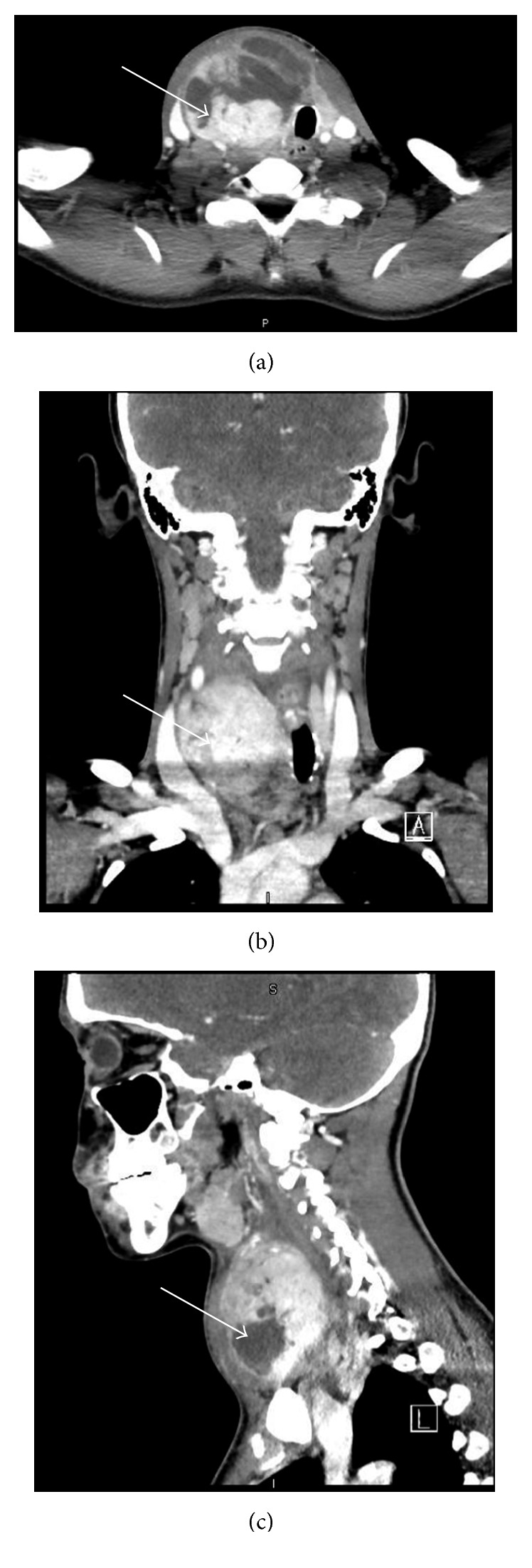
Computed tomography with contrast on admission showed a 6.4 × 6.1 × 7.1 cm heterogeneous mass with mild-to-moderate edema/inflammatory changes and mass effect on the trachea, right internal jugular vein, and right common carotid artery. (a) Cross-sectional, (b) coronal, and (c) sagittal sections.

On admission, the patient was febrile to 100.9°F and tachycardic to 130 beats/minute, with blood pressure of 134/88 mm·Hg, respiratory rate of 18 breaths/minute, and oxygen saturation of 99% on room air. Bedside incision and drainage of the left forearm abscess was performed, and cultures grew methicillin-sensitive *Staphylococcus aureus* (MSSA). Laboratory workup was significant for an elevated white blood cell count of 23.8/*μ*L with differential showing 84.6% neutrophils. Thyroid function tests showed a TSH of 0.02 U/mL, free T4 of 3.89 ng/dL, and free T3 of 5.1 pg/mL, consistent with thyrotoxicosis. The patient was HIV negative and hepatitis C antibody positive. Within the first 24 hours, one out of two blood cultures was positive for MSSA. Computed tomography (CT) scan of the neck with contrast showed a 6.4 × 6.1 × 7.1 cm mass regional to the right thyroid lobe, heterogeneous in appearance with multiple septations and with solid enhancing and cystic components (Figure 1). Mild-to-moderate edema/inflammatory changes were evident along the retropharyngeal space. Additionally, mild luminal narrowing and posterior displacement of the right common carotid artery, lateral displacement and marked narrowing of the right internal jugular vein, and leftward deviation of the trachea were apparent.

**Figure 2:**
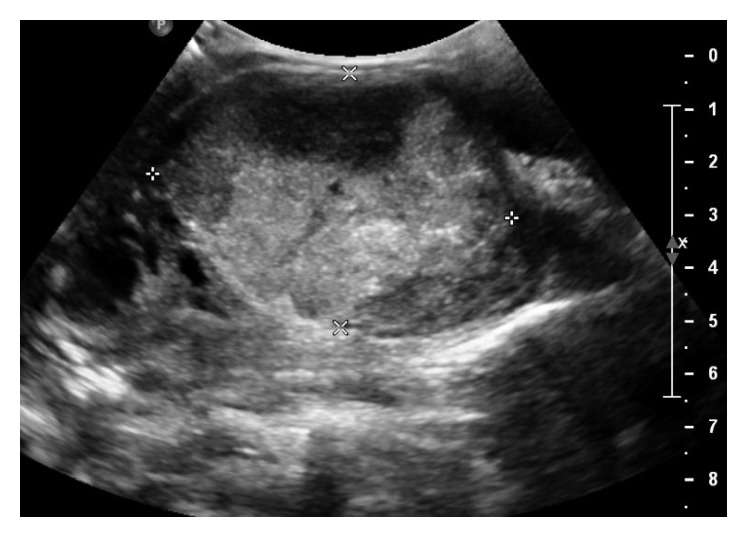
Thyroid ultrasound on hospital day 4 showed a 6.8 × 4.8 × 4.5 cm heterogeneous, necrotic, and hypervascular mass in a sagittal view of the right lobe.

The patient was started on IV ampicillin-sulbactam, as her clinical picture was concerning for AST. Additionally, she was started on naproxen 500 mg twice daily and propranolol 20 mg three times daily for inflammation and thyrotoxicosis, respectively. Over the first four days of hospitalization, the patient remained afebrile, and the leukocytosis and neck swelling improved. A thyroid ultrasound was performed on day 4 to further evaluate for a drainable abscess given the concern for AST. The ultrasound showed a heterogeneous, necrotic, and hypervascular mass in the right lobe measuring 6.8 × 4.8 × 4.5 cm, without any drainable abscess (Figure 2). The patient subsequently underwent ultrasound-guided FNA; cultures grew MSSA, and cytology was consistent with follicular lesion of undetermined significance. A transesophageal echocardiogram ruled out endocarditis. On day 8 of hospitalization, the patient was transitioned from IV ampicillin-sulbactam to oral amoxicillin-clavulanate and discharged home to complete a total of 14 days of antibiotics. She was also discharged on the same regimen of propranolol and naproxen. The patient was instructed to follow up at the endocrinology clinic in one month and receive a repeat FNA in three to six months.

## 3. Discussion

Here, we present a case of AST that could have been misdiagnosed and improperly treated as subacute thyroiditis. Both AST and subacute thyroiditis may present with painful thyroid swelling, fever, and leukocytosis following a URTI. In this case, atypical features for AST include the patient's age, presence of thyrotoxicosis, lack of dysphagia, dysphonia, and airway compromise, and marked clinical improvement within four days of admission [4]. Thyrotoxicosis only occurs in 5–10% of patients with AST and is more often associated with subacute thyroiditis. Subacute thyroiditis, while far more common, is self-limiting; AST, while rare, can be life-threatening. Nevertheless, the patient had several risk factors for AST that increased clinical suspicion, allowing for an appropriate diagnostic workup and course of antibiotics to prevent complications. Misdiagnosis is particularly dangerous as administration of prednisone, used for subacute thyroiditis, may lead to deterioration of AST [10].

In adults, proposed routes of infection for AST include lymphatic or hematogenous spread, direct inoculation of the thyroid or surrounding anatomy, direct extension of an abscess, and spread through a pyriform sinus fistula, usually in the setting of preexisting thyroid disease or an immunocompromised state [2, 11]. Based on the patient's history, several mechanisms may explain her AST. She was an IV drug user who presented with an MSSA abscess in her forearm, along with MSSA bacteremia. Thus, there is the strong possibility for hematogenous spread of MSSA from the forearm abscess to the thyroid. Although the patient denied injecting heroin into her neck or shoulders, there is also the possibility for direct inoculation of the thyroid. Furthermore, the patient had a preexisting multinodular goiter of one-year duration that showed follicular lesion of undetermined significance on cytology. Abnormal thyroid structures, such as multinodular goiters, nodules, or malignancies, have been postulated to make the thyroid gland more susceptible to suppuration [2]. In particular, Erdamar et al. found that the enzymatic free radical defense system was impaired in 41 patients with multinodular goiters and papillary carcinomas [12]. A weakened antioxidant defense mechanism may predispose the thyroid to bacterial infections. Few other cases in the literature report AST in the context of a preexisting multinodular goiter [13–15].

Given the potentially life-threatening sequelae of AST, the threshold for performing ultrasound-guided FNA to rule in/out AST should be relatively low, especially in a patient with one or more predisposing factors. Ideally, thyroid FNA cultures should be obtained prior to antibiotic administration to prevent unnecessary treatment and reduce the risk of a false-negative thyroid culture. Nevertheless, the patient in this case was treated empirically with ampicillin-sulbactam prior to obtaining a thyroid FNA a few days into her hospital stay. Had ampicillin-sulbactam not been started early on in her hospital stay, the thyroid mass may have further compressed the trachea, leading to significant narrowing and airway compromise.

Because AST is rare and may present with varying levels of severity, there are few high-level evidence studies that clearly define optimal management strategies. AST was traditionally managed with a combination of surgical and medical treatment modalities involving partial or total thyroidectomy or surgical drainage and targeted antibiotic therapy. In more recent nonrandomized clinical practice and case reports, there has been a greater emphasis on an upfront medical approach with targeted IV antibiotic therapy [8, 16]. The optimal duration of IV antibiotic therapy is unclear, with expert opinion recommending 14 days [2]. Isolated case reports have continued IV antibiotics for up to 37 days, even in patients who demonstrated early response [17]. As our patient lacked signs of airway compromise and a drainable thyroid abscess, and improved rapidly over the first four days, the decision was made by day 8 to transition her to oral antibiotics and discharge on a 14-day total course. Further studies are needed to refine therapeutic approaches based on the severity of disease presentation.

## 4. Conclusion

This case demonstrates the importance of assessing patient risk factors for AST, even when clinical presentation may seem more typical of subacute thyroiditis. Prompt diagnosis with ultrasound-guided FNA and cultures may allow for targeted antibiotic therapy and prevent complications associated with AST. There is a lack of high-quality studies assessing the optimal management strategy for AST; as such, treatment should be guided by the severity of disease presentation. Further investigation is needed to assess the effectiveness of shorter courses of therapy and/or transition to oral antibiotics in less severe presentations showing early clinical improvement.

## References

[1] Al-Dajani N., Wootton S. H. (2007). Cervical lymphadenitis, suppurative parotitis, thyroiditis, and infected cysts. *Infectious Disease Clinics of North America*.

[2] Paes J. E., Burman K. D., Cohen J. (2010). Acute bacterial suppurative thyroiditis: a clinical review and expert opinion. *Thyroid*.

[3] Hendrick J. W. (1957). Diagnosis and management of thyroiditis. *Journal of the American Medical Association*.

[4] Pearce E. N., Farwell A. P., Braverman L. E. (2003). Thyroiditis. *New England Journal of Medicine*.

[5] Seo J. H., Park Y. H., Yang S. W., Kim H. Y. (2014). Refractory acute suppurative thyroiditis secondary to pyriform sinus fistula. *Annals of Pediatric Endocrinology & Metabolism*.

[6] Parida P. K., Gopalakrishnan S., Saxena S. K. (2014). Pediatric recurrent acute suppurative thyroiditis of third branchial arch origin–our experience in 17 cases. *International Journal of Pediatric Otorhinolaryngology*.

[7] Cieszynski L., Sworczak K., Babinska A., Sledzinski Z. (2013). Recurrent acute suppurative thyroiditis due to pyriform sinus fistula in an adult–case report. *Endokrynologia Polska*.

[8] Ilyin A., Zhelonkina N., Severskaya N., Romanko S. (2007). Nonsurgical management of thyroid abscess with sonographically guided fine needle aspiration. *Journal of Clinical Ultrasound*.

[9] Jacobs A., Gros D. A., Gradon J. D. (2003). Thyroid abscess due to *Acinetobacter calcoaceticus*: case report and review of the causes of and current management strategies for thyroid abscesses. *Southern Medical Journal*.

[10] Spitzer M., Alexanian S., Farwell A. P. (2012). Thyrotoxicosis with post-treatment hypothyroidism in a patient with acute suppurative thyroiditis due to porphyromonas. *Thyroid*.

[11] N’Gattia K. V., Kacouchia N. B., Vroh B. T., Kouassi-Ndjeundo J. (2015). Suppurative thyroiditis and HIV infection. *European Annals of Otorhinolaryngology, Head and Neck Diseases*.

[12] Erdamar H., Cimen B., Gülcemal H., Saraymen R., Yerer B., Demirci H. (2010). Increased lipid peroxidation and impaired enzymatic antioxidant defense mechanism in thyroid tissue with multinodular goiter and papillary carcinoma. *Clinical Biochemistry*.

[13] Akuzawa N., Yokota T., Suzuki T., Kurabayashi M. (2017). Acute suppurative thyroiditis caused by *Streptococcus agalactiae* infection: a case report. *Clinical Case Reports*.

[14] Sicilia V., Mezitis S. (2006). A case of acute suppurative thyroiditis complicated by thyrotoxicosis. *Journal of Endocrinological Investigation*.

[15] Unluturk U., Ceyhan K., Corapcioglu D. (2014). Acute suppurative thyroiditis following fine-needle aspiration biopsy in an immunocompetent patient. *Journal of Clinical Ultrasound*.

[16] Miyauchi A., Inoue H., Tomoda C., Amino N. (2009). Evaluation of chemocauterization treatment for obliteration of pyriform sinus fistula as a route of infection causing acute suppurative thyroiditis. *Thyroid*.

[17] Igarashi H., Yoshino H., Hijikata M. (2017). Acute suppurative thyroiditis in infected thyroid cyst in an adult patient under hemodialysis. *Clinical Case Reports*.

